# Past, present, and future climate space of the only endemic vertebrate genus of the Italian peninsula

**DOI:** 10.1038/s41598-021-01492-z

**Published:** 2021-11-12

**Authors:** Loredana Macaluso, Andrea Villa, Giorgio Carnevale, Massimo Delfino

**Affiliations:** 1 Dipartimento di Scienze della Terra, Università degli Studi di Torino, Turin, Italy; 2 Institut Català de Paleontologia Miquel Crusafont, Universitat Autònoma de Barcelona, Edifici ICTA-ICP, Barcelona, Spain

**Keywords:** Biogeography, Climate-change ecology, Ecological modelling, Evolutionary ecology, Palaeoecology, Palaeontology

## Abstract

The two extant *Salamandrina* species represent a unique case of morphology, ecology, and ethology among urodeles. The range of this genus is currently limited to Italy, where it represents the only endemic vertebrate genus, but its past range extended over a much broader area of Europe, including the Iberian and Balkan peninsulas. ENM analyses using modern occurrences of *Salamandrina* demonstrate that the current climate of the majority of Europe, and especially areas where fossils of this genus were found, is currently not suitable for this genus, neither was it suitable during the last 3.3 million years. This result allows possible assumptions about the climatic influence on the former extirpation of this salamander from several areas of Europe. Furthermore, it shows that, during Pliocene–Pleistocene climatic oscillations, Mediterranean peninsulas, despite being generally considered together because of similar latitude, had different potential to effectively become glacial refugia for this salamander, and possibly for other species as well. Future projections using different CO_2_ emission scenarios predict that climatic suitability will be even more drastically reduced during the next 50 years, underlining once more the importance of conservation strategies and emission-reducing policies.

## Introduction

**Figure 1:**
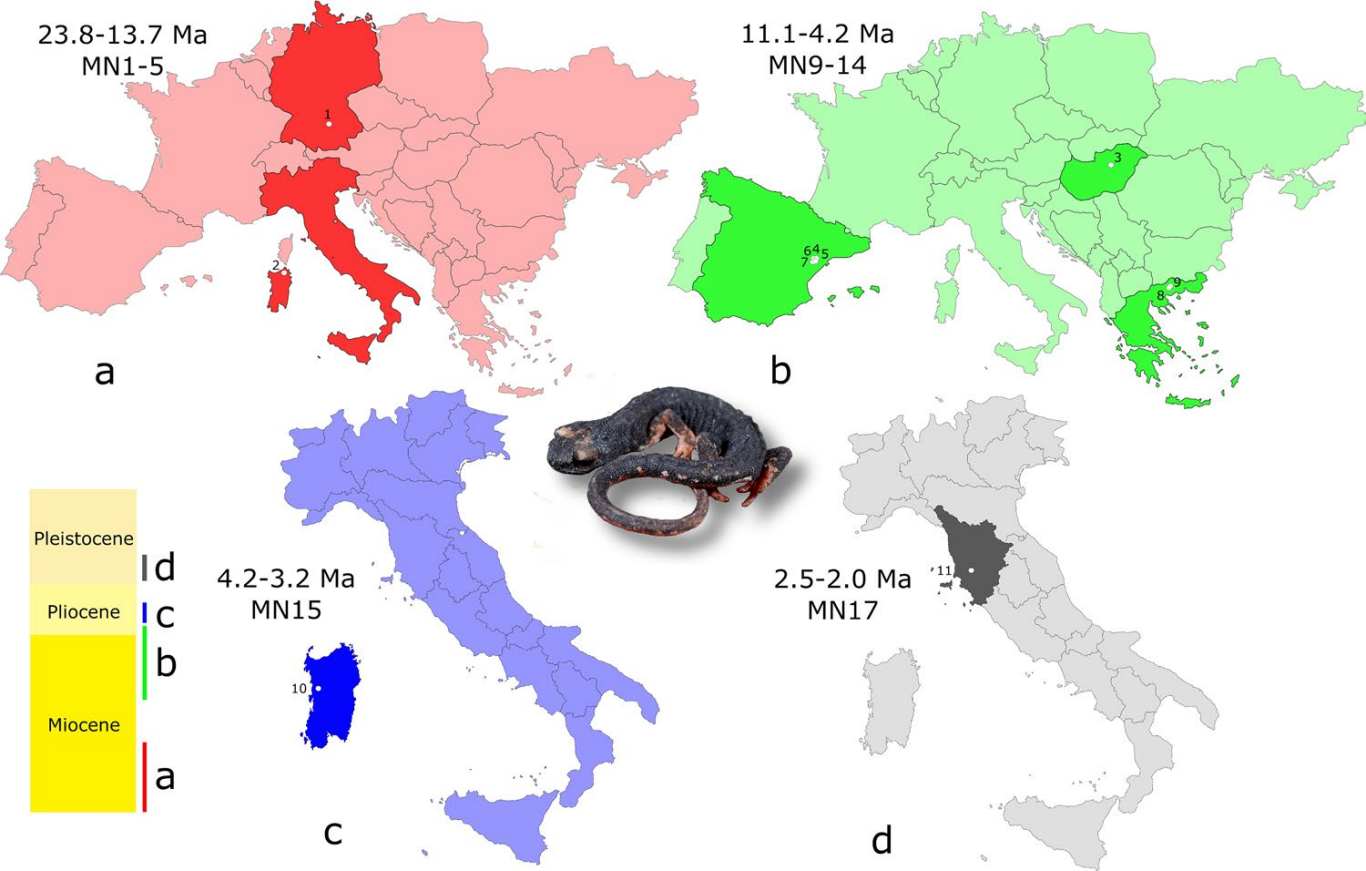
Time line of fossil localities yielding remains attributed to *Salamandrina* sp. (data from Macaluso et al*.*^13^) from early Miocene (**a**) and late Miocene to early Pliocene (**b**) of Europe, and from Pliocene (**c**) and Pleistocene (**d**) of Italy. Information about the age in the left above corner of each map. “MN” refers to Mammal Neogene Units (see Agustí et al.^16^ for the definition of the units). 1—Petersbuch, Germany (MN 4); 2—Oschiri, Italy (MN 1–5); 3—Felsötárkány Basin, Hungary (MN 9); 4—La Gloria 11, Spain (MN 10); 5—La Roma 1 and 2, Spain (MN 10); 6—Masada Ruea 2, Spain (MN 11); 7—Puente Minero 3, Spain (MN 11); 8—Ano Metochi 3, Greece (MN 13); 9—Maramena, Greece (MN 13/14); 10—Capo Mannu D1 Local Fauna, Italy (MN 15); 11—Montagnola Senese, Italy (MN 17). Current distribution visible in Fig. 3A. Picture of *Salamandrina* by E. Razzetti.

Climatic oscillations during both the Pliocene–Pleistocene transition and the Pleistocene widely influenced the geographic patterns of species occurring in temperate zones^1–3^. Northern Europe was affected by unfavourable glacial conditions for temperate animal and plant species that were driven to southern refugia in the three Mediterranean peninsulas. As a result, the Balkan, Italian, and Iberian peninsulas currently show high genetic diversity and endemism^4,5^. However, at a fine resolution, several contrasting patterns can be recognized by comparing species richness and endemism of ectothermic tetrapods from these three geographic areas. In particular, the Italian biogeographic Province (as defined by Lanza and Corti^6^) has a remarkably low proportion of endemic reptiles (17%), and there are no endemic genera, with the few endemic species being mainly restricted to islands^7^. Conversely, the same area has the most diversified amphibian fauna of the Mediterranean Region and hosts the greatest number of endemic taxa—approximately 50% of the Italian amphibian species are endemic^8,9^. Moreover, looking at the fossil record of extant reptiles, there is no evidence for a range shrinkage of taxa; which is to say that no taxon that was formerly widespread in Europe, now exclusively survives in the Italian biogeographic Province. Conversely, fossil remains attributed to the Italian endemic urodele genera *Salamandrina* and *Speleomantes* were reported from areas of Europe out from their current range. While the European fossil record of the latter is extremely poor (limited to a single trunk vertebra from the middle Miocene of Slovakia^10^), remains of *Salamandrina* were described from the Miocene of Hungary, Germany, and Spain, and from the late Miocene and Mio-Pliocene transition of Greece^11–13^. From Pliocene on, *Salamandrina* was only reported from Italy (see Fig. 1 for a visual time line of the evolutionary history of this genus), in particular from the Pliocene of Sardinia (Italy)^14^, and from the Early Pleistocene of peninsular Italy^13^. The current geographic ranges of the only two species of *Salamandrina* is limited to the Italian peninsula, where they represent the only endemic vertebrate genus^15^, suggesting an extirpation of this genus from most Europe, including the Iberian and Balkan peninsulas. The colonization of peninsular Italy by this genus is, at least apparently, a rather recent event, given that no pre-Quaternary fossils are known from the relatively-well scrutinized palaeoherpetological assemblages of the area^13^.

It is well known that climate is one of the main abiotic variables that exerts a considerable influence on shaping the distribution of species in space and time^17,18^. A recent study performed by Iannella et al*.*^19^ confirmed that, like other taxa, the two *Salamandrina* species found a refugium in small regions of Central and Southern Italy during the Pliocene–Pleistocene glaciation cycles. However, in this context, the reasons for, and timing of, the extirpation of *Salamandrina* from the Iberian and Balkan peninsulas are unknown and it remains unclear why only the Italian peninsula acted as a glacial refugium. The purpose of this work is to understand whether the European areas forming the past geographic range of *Salamandrina* are currently, or were during selected time bins of Pleistocene and Pliocene, climatically suitable for these salamanders. Herein, we use the extensively-applied Ecological Niche Modelling (ENM) methodology (generally used to reconstruct the potential niche of extant and extinct taxa^20–24^) to investigate how the climate of Europe evolved through time and to assess if the climate of the Italian peninsula is/was particularly suitable for *Salamandrina*, especially in comparison with the Iberian and Balkan peninsulas. Based on predicted future CO_2_ emission patterns^25,26^, we subsequently project the model on different future scenarios to predict the possible suitability of future habitats of the two *Salamandrina* species (*Salamandrina perspicillata* and *Salamandrina terdigitata*) throughout the Italian peninsula.

## Material and methods

The modern distribution of *Salamandrina* was composed by compiling all of the available records of the two extant species available in the atlas database of the *Societas Herpetologica Italica* (SHI) and from the online depositories GBIF.org (10.15468/dl.as6sk2)^27^ and is formed by 605 occurrence data points in total. Nineteen bioclimatic variables (see Appendix S1 for a complete list) for the modern climate scenario were downloaded from PaleoClim^28^. Pearson's pairwise correlation coefficients were calculated with R^29^ to measure the autocorrelation between the variables. To reduce co-linearity between variables and prevent over-fitting, we retained the combinations of climate variables with a Pearson’s coefficient of less than 0.75 (see Appendix S2 and S3). Six independent variables were selected: temperature seasonality, mean temperature of wettest quarter, mean temperature of driest quarter, mean temperature of the warmest quarter, precipitation seasonality, precipitation of wettest quarter, and precipitation of warmest quarter. The same independent bioclimatic variables were downloaded for different time bins:

- Last Glacial Maximum (LGM; ca. 21,000 years BP)^30^;
- Last Interglacial (LIG; ca. 130,000 years BP)^31^;
- MIS 19 of Pleistocene (ca. 787,000 years BP)^28^;
- mid-Pliocene warm period (3.205 million years ago)^32^;
- Marine Isotope Stage M2 of Pliocene (ca. 3.3 million years ago)^33^;
- 2070 (average for 2061–2080), deriving from climate projections from different GCMs (see Appendix S4) and four representative concentration pathways (RCPs) for the greenhouse gas scenarios corresponding to four Shared Socio-economic Pathways (SSPs; 2.6, 4.5, 6.0, 8.5)^25,26^.

Based on the considerations of Sillero and Barbosa^34^, comparable resolution between data points and climatic data were obtained downloading files with resolution of 2.5 min. The ecological niche of *Salamandrina* and past and future projections of climatic suitability were derived using the Ensemble Modelling approach through ‘biomod2’ package of R^35^. More in detail, we implemented three ENM methods: the Generalized Linear Model^36^, randomForest^37^ and MaxEnt v. 3.4.1^38^. The map obtained as output from the software shows the so-called potential climatic niche was modified using QGIS 3.12.1^39^, representing an explicitly predictive spatial map of the current geographical location of climatic spaces suitable for these taxa^40^. Seventy percent of data were used to train the model and the remaining thirty percent was used for testing. Model performance was assessed using the true skill statistic (TSS), the evaluation scores of which range from -1 to 1, where a score of 1 demonstrates perfect model performance and values lower than 0 are considered worse than a random model^41^.

## Results

**Figure 2:**
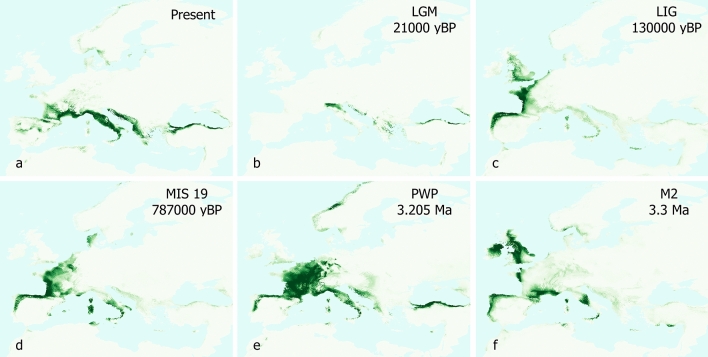
ENM of *Salamandrina* at the European scale: model for present day (**a**) and projections of the model on different time bins, from Pleistocene (**b**–**d**) to Pliocene (**e**,**f**). Climatic suitability is reported on a scale between 0 (white) and 1 (darkest green). It is worth noting that Italy is the Mediterranean peninsula showing the most temporally extended areas of suitable climate (dark green) for *Salamandrina*, whereas suitable climates are limited in the rest of Europe, and not present at all during Pliocene where the non-Italian fossil localities of Fig. 1 are located. Abbreviations: LGM, Last Glacial Maximum; LIG, Last Interglacial; M2, Marine Isotope Stage M2 of Pliocene; PWP, mid-Pliocene Warm Period. Maps modified with QGIS 3.12.1^39^.

The ENM run at European-scale using ‘biomod2’ resulted in the ensemble model reported in Fig. 2a, in which darker green shows areas with better-predicted conditions. Based on TSS for testing data (0.977; see also Appendix S5), the model is well-supported. Projections on past climate scenarios are reported in Fig. 2b–f.

**Figure 3:**
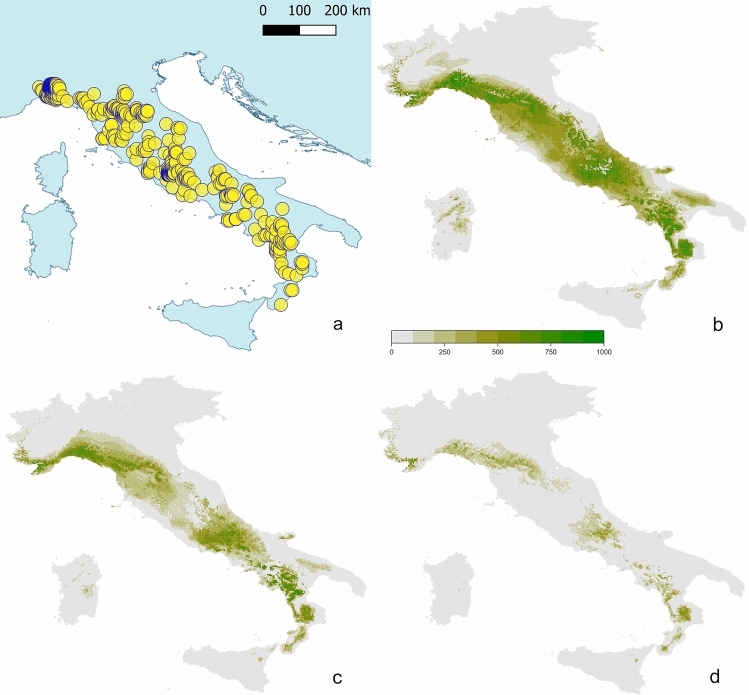
Extant distribution and future suitability projections for *Salamandrina* spp. in Italy. (**a**) Presence data of *Salamandrina* spp. used in this analysis defining the current range of the species; (**b**) Ecological Niche Modelling for *Salamandrina* in Italy using extant bioclimatic variables. (**c**,**d**) Future projections using the bioclimatic variables for 2070: c, RCP 2.6, very stringent pathway (1 °C mean global warming increase during 2046–2065); d, RCP 8.5, very relaxed pathway (2 °C mean global warming increase during 2046–2065). Progressively darker green indicates higher climatic suitability for *Salamandrina* in a scale between 0 and 1000.

The modern occurrences of *Salamandrina* are reported in Fig. 3a with a representation of the current Italian scenario obtained with ‘biomod2’ (TSS for testing data = 0.634, cutoff = 330, sensitivity = 88.67, specificity = 74.7; Fig. 3b), followed by future projections using General Circulation Model CCSM4 with the representative concentration pathways 2.6 and 8.5 (Fig. Fig. 3c,d respectively). We herein reported the best and worst scenario possible using one of the different GCMs used for the future projections, considering that projections based on other GCMs can be slightly different concerning the zones that are characterised by reduction of suitability, but they generally share a similar pattern of increasing of climatically unsuitable areas (representations of the future projections obtained using others GCMs and RCPs are available in Appendix S4).

## Discussion

Our results show that very few areas of Europe seem to be equally as suitable for *Salamandrina* as the Italian peninsula (Fig. 2). All the non-Italian localities that yielded fossils referable to this genus are currently characterized by low suitability and the same pattern is more accentuated when the model is projected on past scenarios (Fig. 2b–f), with the climatic suitability for *Salamandrina* being even more reduced. The climatic suitability on the Mediterranean island of Sardinia during LGM is definitely lower than in peninsular Italy, but in the other time bins it does not appear to be significantly different. The last fossil remains attributed to *Salamandrina* from this island is from the early Pliocene of Capo Mannu D1 Local Fauna (dated to 4.2–3.2 Ma)^14^. The projection of our model on comparable Pliocene intervals (Fig. 2e,f) shows that climate was at that time suitable for this urodele in Sardinia, and its extirpation could be linked to subsequent prolonged unsuitable condition during glacial maxima (as shown by the low suitability during LGM; Fig. 2b) of Plio-Pleistocene.

As far as continental populations are concerned, there are several examples of European organisms whose geographic range was subject to a southern shift due to Plio-Pleistocene climatic oscillations^42–45^. The extirpation of *Salamandrina* from central Europe (where its past occurrence is documented by the German and Hungarian Miocene localities) might be linked to similar events, and our analyses supports this hypothesis, considering that the model herein presented shows that the climate of this area was poorly suitable for *Salamandrina* during mid-Pliocene (Fig. 2e) and even less suitable during the cooling Pliocene event M2 (Fig. 2f). However, the last fossil occurrence from Central Europe is from the late Miocene of Hungary (from 11.1 to 9.7 Ma; Fig. 1b)^11^ and therefore too far from time bins herein analysed to convincingly support the hypothesis of an extirpation due to Plio-Pleistocene climatic oscillations. As the fossil record from this area is rather scarce, further findings are needed to shed new light on the puzzling disappearance of *Salamandrina*.

Concerning Southern Europe, the models herein presented demonstrate that although the Mediterranean peninsulas are generally pooled together due to a similar role of refugia during the Pliocene–Pleistocene, because of their comparable latitude and Mediterranean affinity, our analysis demonstrates that Central and Southern Italy theoretically had the climatic potential to act as a better refugium for *Salamandrina*. In the case of this taxon, a direct link between Pliocene–Pleistocene glaciation cycles and its extirpation does not exist because according to the currently known fossil record there is no direct evidence of a Pliocene geographic range of *Salamandrina* outside the Italian territory. The last occurrence of this genus outside Italy is from the late Miocene and Miocene-Pliocene transition of Greece (Ano Metochi and Maramena, respectively; MN 13–14; Fig. 1b), which is slightly more recent than the Spanish occurrences from the Alfambra-Teruel Basin (MN 10–11)^13^. The projection on Pliocene time bins shows that the climatic suitability of the Balkan and Iberian peninsulas was low since 3.3 Ma (MN 15–16). The west coast of the Balkan peninsula occasionally shows a higher suitability (e.g., during the present day and the mid-Pliocene Warm Period) compared to the east coast, but the presence of *Salamandrina* has never been reported from this area (neither as extant populations nor as fossil specimens). The east part of Spain (the area yielding *Salamandrina* remains) shows a very low suitability, almost zero during LIG and LGM time bins, and very limited during MIS 19, mid-Pliocene Warm Period, and Pliocene M2 time bins. As we cannot exclude the possibility that this genus was already extirpated from most of Europe before M2 and PWP time bins, the roots of the extirpation from the Balkan and Iberian peninsulas might be found in changes happened earlier than these latter, explored as follows using the climatic preference of *Salamandrina* (Appendix S6). As a matter of fact, the annual mean temperature (based on rodent associations) reconstructed for the Alfambra-Teruel Basin during MN 10–11 and for the Greek localities during MN13/14 (including Ano Metochi) are respectively 12.6 ± 4.8 °C and 14.9 ± 4.8 °C^45^, perfectly in between the climatic space of the extant *Salamandrina* (see Appendix S6). Concerning subsequent time bins of Spain, although the reconstructed paleotemperature of the localities pertaining to time intervals ranging between MN 11 and MN 13 are within the extant optimal range for *Salamandrina*^46^ (with the extreme case of Salobreña near Granada, with a value of 16 ± 4.8 °C, corresponding to the upper quartile of the Gaussian in Appendix S6), the localities corresponding to MN 14 and MN 15 generally show higher values. In particular, the annual mean temperature of La Gloria 4 (MN 14) and the two southern Spanish localities of Belmez and Moreda (both MN 15) is 20.6 ± 4.8 °C^46^. This value is considerably higher than the extant range of annual mean temperatures of the areas inhabited by *Salamandrina*. Therefore, matching these data with the very low suitability shown by this area during M2 and PWP (Fig. 2e–f), it seems likely that an increasingly warmer climate in the Iberian peninsula marked the first steps of the extirpation of this genus from the area during the early Pliocene, before the beginning of the Pliocene–Pleistocene climatic oscillations.

Concerning the Balkan peninsula, palaeobotanical reconstructions showed that vegetation persisted nearly unchanged throughout the Miocene, corresponding to the modern type of laurel forest with deciduous taxa, typical of a winter-dry climatic zone^47^. A marked turnover towards the modern Mediterranean flora of the forest structure occurred during the Pliocene (the same vegetational change was almost coeval in the Iberian peninsula according to Martinetto and Vieira^48^). The Balkan area is characterized by a transition from winter-dry climate during the Miocene to fully humid or summer-dry ones in Pliocene times^47^. *Salamandrina* is currently present in Italy in areas characterized by both these climatic zones (even if its geographic range also includes other climatic categories^49^, and thus there is no significant reason to explain the extirpation of this genus from the Balkans based on these data. However, the areas where *Salamandrina* is expected to suffer the highest decrease in habitat suitability according to our future projections (see below; Fig. 3c,d) are those characterized by an extension of summer-dry climatic zones before 2069 (see Fig. 2c of Jylhä et al*.*^50^). Conversely, Italian areas characterized by other climatic zones are those predicted to preserve the highest suitability in future decades. This could mean that the expansion of the summer-dry climatic zone in Greece, that today is the widest climatic zone on the eastern part of the country^49^, was linked to at least the first steps towards the extirpation of *Salamandrina*.

### Future projections

All the scenarios dealing with the future suitability for *Salamandrina* species, using different GCMs and RCPs (Fig. 3c,d, Appendix S4), converge to the future reduction of climatic suitability before 2070, particularly in Central Italy. The north and south extremes of their cumulative geographic range seem to be less affected by future climatic change, but different RCPs yielded substantially different scenarios. All the RCPs are considered plausible, depending on the volume of greenhouse gases emitted in the years to come. RCPs range from a very optimistic pathway (RCP 2.6), in which mean global warming increases between the years 2046–2065 is limited to 1 °C, to the most catastrophic scenario (RCP 8.5), in which the mean global warming increase during the same period is 2 °C^25^. It is worth noting that RCP 8.5, with a continuous rise in emissions throughout twenty-first century^51^, is currently considered as highly unlikely (due to an overestimation of projected coal outputs; for further details see Rutledge^52^ and Hausfather and Peters^53^). Nonetheless, this last, worst-possible scenario (Fig. 3d), which predicts a drastically-reduced habitat suitability, is substantially different from the best scenario possible (Fig. 3c). This is consistent using different GCMs (in Appendix S4), not only CCSM4 (shown in Fig. 3). Policies devoted to the containment of the anthropically-induced climate change will also be very important to limit the negative impact that predicted warming is going to have on these amphibians. Even the most optimistic scenario proves that human induced climate change will have future negative effects on *Salamandrina*. It is likely that similar, or even less intense, non-human induced climatic changes in the past caused the extirpation of several populations across Europe. The low extinction rates of Pleistocene amphibians (when compared to those of contemporary birds and mammals) could indirectly suggest that the long term survival of amphibian species could depend from even small populations with limited ranges^54^ (as in the case of the populations of *Salamandrina* survived in refugia of Central and Southern Italy^19^); therefore, in the case of these species, conservation efforts at a local scale might be successful for granting a long term survival of a species, if matched with the correct policies at global scale to reduce emissions. Considering the future perspectives reported herein for these small inhabitants of mesophilous or subthermophilous woods^55,56^, each effort to reduce the future global warming has proven once again to be strikingly important. However, given the cited above resilient nature of amphibian species^56^, local governments should pay attention to forest and river management plans in order to favour habitat suitability for these salamanders, such as those suggested by Basile et al*.*^57^. These schemes include maintaining a suitable number of trees with a trunk diameter exceeding 30 cm, the implementation of selective logging along the banks of streams, and leaving an adequate buffer zone around streams. Conservation efforts to limit the reduction of habitat suitability in the last refugium of this genus (i.e., the Italian peninsula) are important given the biological uniqueness of these species. Interestingly, these taxa are strikingly different from other salamandrid species, not only due to their peculiar skeletal anatomy^58^, but also in their external morphology. They display a peculiar ventral colour pattern and are the only salamandrid with four digits on both front and hind limbs^3^. Furthermore, they exhibit a suite of unique behavioural peculiarities, being the only urodele able to perform the unkenreflex (i.e., curling the body dorsally to reveal their ventral colour pattern as a defensive behaviour^59^). In addition, *Salamandrina perspicillata* is the only urodele currently known to show the so-called “stand up” behaviour, standing on its hind limbs and tails to display the ventral side^60^. Because of these morphological and behavioural peculiarities, that make the degree of uniqueness and 'distintinctiveness' of this taxon very high^61^, the presence of the two *Salamandrina* species notably increases not only taxic diversity, but also the morphological disparity of the ecosystems that include them^62^. Therefore, in terms of conservation biology, a responsible and far-sighted wildlife management plan should acknowledge a conservation priority to the irreplaceable areas in which these species occur, even if the region is not considered to be vulnerable in the short term^63–65^.

## Conclusions

The present work analyses the climatic suitability of *Salamandrina* species, and shows that the European areas where fossils attributed to this taxon occurred had low suitability during the M2 (Pliocene), mid-Pliocene Warm Period, MIS 19 (Pleistocene), Last Interglacial, Last Glacial Maximum, and present day. Suitability was particularly low during the Last Glacial Maximum in Sardinia, pointing to glacial maxima as possible climatic drivers towards the extirpation of the genus from this large Mediterranean island. Models herein reported show that climatic suitability of Greece and Spain localities that yielded *Salamandrina* remains was already low since 3.3 Ma, highlighting that despite being generally pooled together as glacial refugia during the Plio-Pleistocene, the Italian peninsula had the climatic potential to act as a better refugium for *Salamandrina* than the Iberian and Balkan peninsulas. However, the stratigraphic range of *Salamandrina* cannot be directly extended to the Pliocene of whole Europe, as the only Plio-Pleistocene remains are from Italian localities. For this reason, the possibility that this genus was already extirpated from Europe during the early Pliocene should not be eliminated, and the lower suitability of the Iberian and Balkan peninsulas compared with the Italian one should be considered as theoretical until the fossil record is more completely known. Climatic changes that took place during the earliest Pliocene could have set the roots to the subsequent extirpation of *Salamandrina* from the Iberian and Balkan peninsulas. In particular, large scale previously detected increasing temperatures in Spain and the expansion of the summer-dry climatic zone in Greece during Pliocene could have represented the first steps towards the extirpation of this genus.

Our analyses also show that a marked reduction of the habitat suitability seems to be inevitable in the future; however, this is strongly dependent on the RCP used for the projection. This once again highlights the importance of cutting CO_2_ emissions during the next few crucial years, and indicates the need to follow precise management plans to achieve conservation objectives in favour of these vulnerable species and their habitat.

## Supplementary Information

Supplementary Information.

## Data Availability

Data supporting the results are available as online Supplementary Material.
