# Supplementary material for: Past, present, and future climate space of the only endemic vertebrate genus of the Italian peninsula
Source: Sci Rep. 2021 Nov 12;11:22139. doi: 10.1038/s41598-021-01492-z (PMC8590061; doi:10.1038/s41598-021-01492-z)
Supplement: Supplementary file 1 — Supplementary Information. [file 41598_2021_1492_MOESM1_ESM.docx]

**Investigating climatic influence on extirpations through models should consider the timing of the last fossil occurrence: a case study**

**Appendix S1.** List of the coded bioclimatic variables from PaleoClim ([https://www.paleoclim.org/](https://www.worldclim.org/)). A quarter is a period of three months (1/4 of the year).

This scheme follows that of ANUCLIM, except that for temperature seasonality the standard deviation was used because a coefficient of variation does not make sense with temperatures between -1 and 1.

BIO1 = Annual Mean Temperature

BIO2 = Mean Diurnal Range (Mean of monthly (max temp - min temp))

BIO3 = Isothermality (BIO2/BIO7) (×100)

BIO4 = Temperature Seasonality (standard deviation of the mean monthly temperature ×100)

BIO5 = Max Temperature of Warmest Month

BIO6 = Min Temperature of Coldest Month

BIO7 = Temperature Annual Range (BIO5-BIO6)

BIO8 = Mean Temperature of Wettest Quarter

BIO9 = Mean Temperature of Driest Quarter

BIO10 = Mean Temperature of Warmest Quarter

BIO11 = Mean Temperature of Coldest Quarter

BIO12 = Annual Precipitation

BIO13 = Precipitation of Wettest Month

BIO14 = Precipitation of Driest Month

BIO15 = Precipitation Seasonality (Coefficient of Variation: ratio of the standard deviation of the monthly total precipitation to the mean monthly total precipitation).

BIO16 = Precipitation of Wettest Quarter

BIO17 = Precipitation of Driest Quarter

BIO18 = Precipitation of Warmest Quarter

BIO19 = Precipitation of Coldest Quarter

**Appendix S2.** Dendrogram based on distance built on Pearson’s correlation (less distance = more correlation).


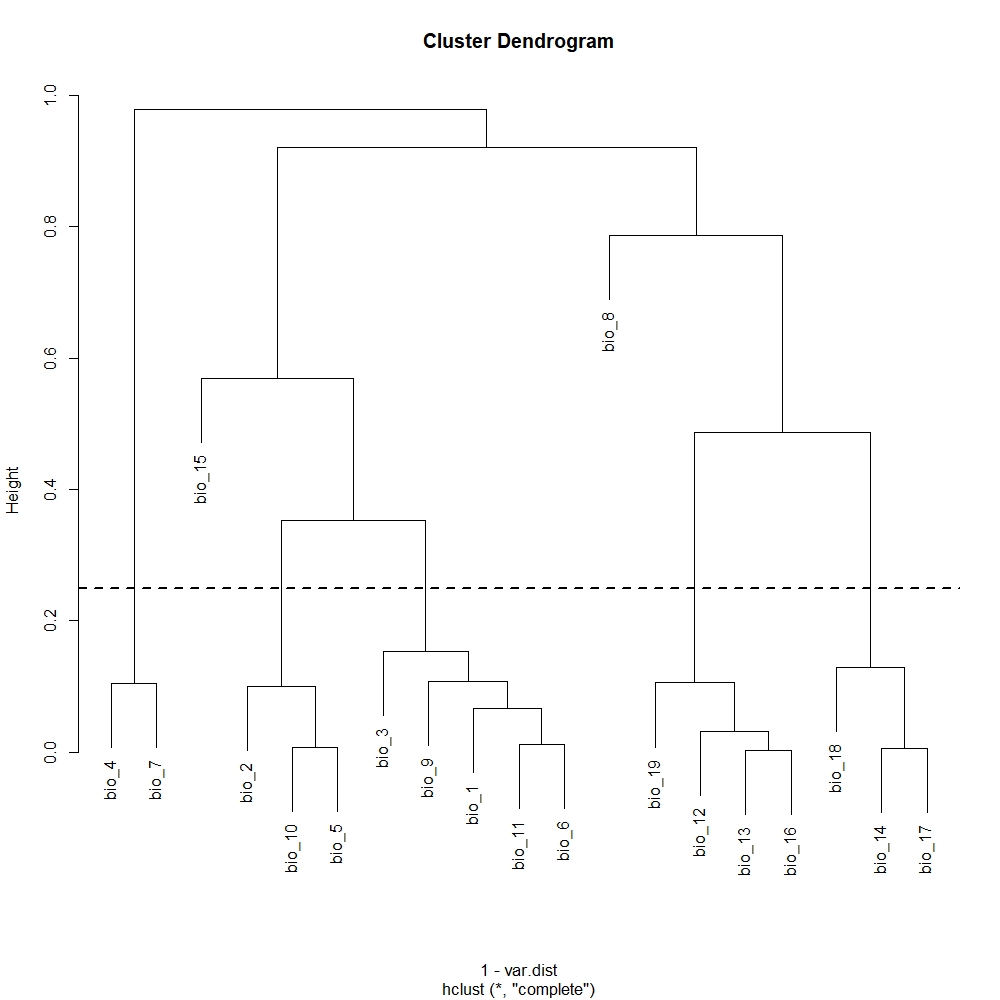


**Appendix S3.** Pearson’s pairwise comparison table.

|  | **pearson.correlation.coefficient.bio_1** | **pearson.correlation.coefficient.bio_10** | **pearson.correlation.coefficient.bio_11** | **pearson.correlation.coefficient.bio_12** | **pearson.correlation.coefficient.bio_13** | **pearson.correlation.coefficient.bio_14** | **pearson.correlation.coefficient.bio_15** | **pearson.correlation.coefficient.bio_16** | **pearson.correlation.coefficient.bio_17** | **pearson.correlation.coefficient.bio_18** | **pearson.correlation.coefficient.bio_19** | **pearson.correlation.coefficient.bio_2** | **pearson.correlation.coefficient.bio_3** | **pearson.correlation.coefficient.bio_4** | **pearson.correlation.coefficient.bio_5** | **pearson.correlation.coefficient.bio_6** | **pearson.correlation.coefficient.bio_7** | **pearson.correlation.coefficient.bio_8** | **pearson.correlation.coefficient.bio_9** | **mean** |
| --- | --- | --- | --- | --- | --- | --- | --- | --- | --- | --- | --- | --- | --- | --- | --- | --- | --- | --- | --- | --- |
| **bio_1** | 1 | 0.958429 | 0.969197 | -0.54866 | -0.52169 | -0.55603 | 0.478073 | -0.52457 | -0.55011 | -0.71214 | -0.30894 | 0.847078 | 0.871682 | -0.46858 | 0.931471 | 0.933426 | -0.10133 | 0.520836 | 0.898573 | 9.460047 |
| **bio_10** | 0.958429 | 1 | 0.861108 | -0.63267 | -0.59772 | -0.63612 | 0.454911 | -0.60256 | -0.63291 | -0.72603 | -0.42521 | 0.899211 | 0.734865 | -0.20014 | 0.992279 | 0.800956 | 0.176091 | 0.614859 | 0.822933 | 19.59333 |
| **bio_11** | 0.969197 | 0.861108 | 1 | -0.43675 | -0.4179 | -0.45304 | 0.468954 | -0.41902 | -0.44343 | -0.66086 | -0.17835 | 0.742984 | 0.926276 | -0.66996 | 0.821105 | 0.988464 | -0.33257 | 0.391467 | 0.915166 | -0.66606 |
| **bio_12** | -0.54866 | -0.63267 | -0.43675 | 1 | 0.968385 | 0.907235 | -0.38395 | 0.976023 | 0.926558 | 0.829363 | 0.89455 | -0.70355 | -0.42412 | -0.08557 | -0.63875 | -0.33803 | -0.37411 | -0.44211 | -0.45281 | 499.8089 |
| **bio_13** | -0.52169 | -0.59772 | -0.4179 | 0.968385 | 1 | 0.802916 | -0.23475 | 0.996672 | 0.825916 | 0.778282 | 0.899888 | -0.6662 | -0.40693 | -0.07126 | -0.60067 | -0.32364 | -0.34336 | -0.41951 | -0.42387 | 64.55867 |
| **bio_14** | -0.55603 | -0.63612 | -0.45304 | 0.907235 | 0.802916 | 1 | -0.56232 | 0.814135 | 0.993962 | 0.876342 | 0.709611 | -0.70304 | -0.43676 | -0.05841 | -0.65169 | -0.36026 | -0.3591 | -0.35262 | -0.52523 | 22.93672 |
| **bio_15** | 0.478073 | 0.454911 | 0.468954 | -0.38395 | -0.23475 | -0.56232 | 1 | -0.25285 | -0.55891 | -0.50707 | -0.1701 | 0.440796 | 0.462069 | -0.24348 | 0.449578 | 0.431745 | -0.02133 | 0.204995 | 0.503217 | 37.68456 |
| **bio_16** | -0.52457 | -0.60256 | -0.41902 | 0.976023 | 0.996672 | 0.814135 | -0.25285 | 1 | 0.836183 | 0.784036 | 0.906778 | -0.67197 | -0.40771 | -0.07632 | -0.60597 | -0.32428 | -0.34964 | -0.42961 | -0.42492 | 176.2738 |
| **bio_17** | -0.55011 | -0.63291 | -0.44343 | 0.926558 | 0.825916 | 0.993962 | -0.55891 | 0.836183 | 1 | 0.871595 | 0.738422 | -0.70382 | -0.43002 | -0.07206 | -0.64732 | -0.34779 | -0.37146 | -0.36606 | -0.50766 | 77.47311 |
| **bio_18** | -0.71214 | -0.72603 | -0.66086 | 0.829363 | 0.778282 | 0.876342 | -0.50707 | 0.784036 | 0.871595 | 1 | 0.51305 | -0.7554 | -0.64357 | 0.205739 | -0.7254 | -0.58967 | -0.12266 | -0.21338 | -0.75619 | 138.7585 |
| **bio_19** | -0.30894 | -0.42521 | -0.17835 | 0.89455 | 0.899888 | 0.709611 | -0.1701 | 0.906778 | 0.738422 | 0.51305 | 1 | -0.51904 | -0.17179 | -0.27631 | -0.43895 | -0.08009 | -0.48062 | -0.50973 | -0.1337 | 117.6282 |
| **bio_2** | 0.847078 | 0.899211 | 0.742984 | -0.70355 | -0.6662 | -0.70304 | 0.440796 | -0.67197 | -0.70382 | -0.7554 | -0.51904 | 1 | 0.739811 | -0.11959 | 0.925459 | 0.647261 | 0.310735 | 0.507718 | 0.74991 | 9.971503 |
| **bio_3** | 0.871682 | 0.734865 | 0.926276 | -0.42412 | -0.40693 | -0.43676 | 0.462069 | -0.40771 | -0.43002 | -0.64357 | -0.17179 | 0.739811 | 1 | -0.71199 | 0.708835 | 0.908483 | -0.36812 | 0.286994 | 0.845954 | 31.3563 |
| **bio_4** | -0.46858 | -0.20014 | -0.66996 | -0.08557 | -0.07126 | -0.05841 | -0.24348 | -0.07632 | -0.07206 | 0.205739 | -0.27631 | -0.11959 | -0.71199 | 1 | -0.13579 | -0.73517 | 0.894592 | 0.138489 | -0.56086 | 827.3414 |
| **bio_5** | 0.931471 | 0.992279 | 0.821105 | -0.63875 | -0.60067 | -0.65169 | 0.449578 | -0.60597 | -0.64732 | -0.7254 | -0.43895 | 0.925459 | 0.708835 | -0.13579 | 1 | 0.752975 | 0.257077 | 0.610016 | 0.801104 | 26.49263 |
| **bio_6** | 0.933426 | 0.800956 | 0.988464 | -0.33803 | -0.32364 | -0.36026 | 0.431745 | -0.32428 | -0.34779 | -0.58967 | -0.08009 | 0.647261 | 0.908483 | -0.73517 | 0.752975 | 1 | -0.44236 | 0.341433 | 0.891958 | -6.01903 |
| **bio_7** | -0.10133 | 0.176091 | -0.33257 | -0.37411 | -0.34336 | -0.3591 | -0.02133 | -0.34964 | -0.37146 | -0.12266 | -0.48062 | 0.310735 | -0.36812 | 0.894592 | 0.257077 | -0.44236 | 1 | 0.329956 | -0.2181 | 32.51165 |
| **bio_8** | 0.520836 | 0.614859 | 0.391467 | -0.44211 | -0.41951 | -0.35262 | 0.204995 | -0.42961 | -0.36606 | -0.21338 | -0.50973 | 0.507718 | 0.286994 | 0.138489 | 0.610016 | 0.341433 | 0.329956 | 1 | 0.180826 | 13.55825 |
| **bio_9** | 0.898573 | 0.822933 | 0.915166 | -0.45281 | -0.42387 | -0.52523 | 0.503217 | -0.42492 | -0.50766 | -0.75619 | -0.1337 | 0.74991 | 0.845954 | -0.56086 | 0.801104 | 0.891958 | -0.2181 | 0.180826 | 1 | 7.27389 |

**Appendix S4.** Future projections for 2070 performed using R package ‘biomod2’, under different GCMs. GCMs: BC = BCC-CSM1-1, CE = CESM1-CAM5-1-FV2, CN = CNRM-CM5, GF = GFDL-CM3, GD = GFDL-ESM2G, GS = GISS-E2-R, HD = HadGEM2-AO, HE = HadGEM2-ES, IP = IPSL-CM5A-LR, MI = MIROC-ESM-CHEM, MR = MIROC-ESM, MC = MIROC5, MG = MRI-CGCM3, NO = NorESM1-M. See www.worldclim.org for details about the models. RCPs: 45 = 4.5, 26 = 2.6 (see main text).


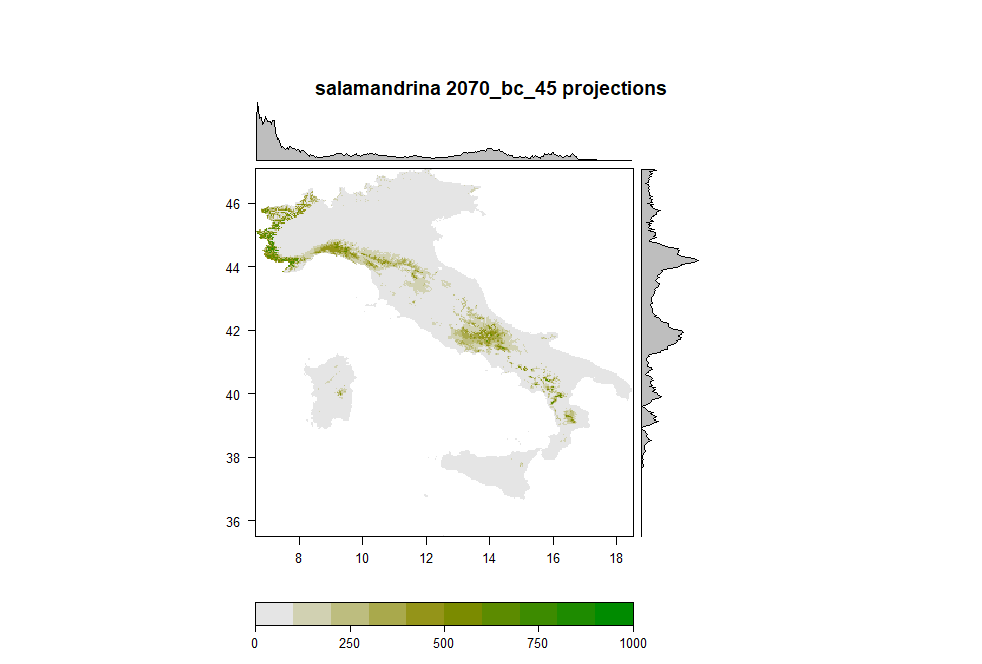

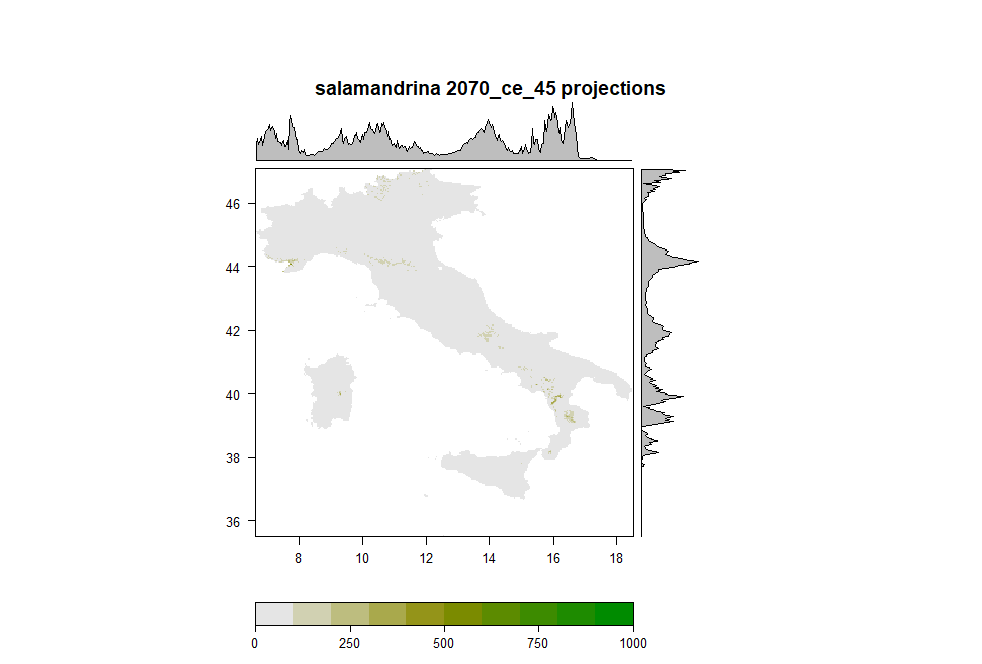

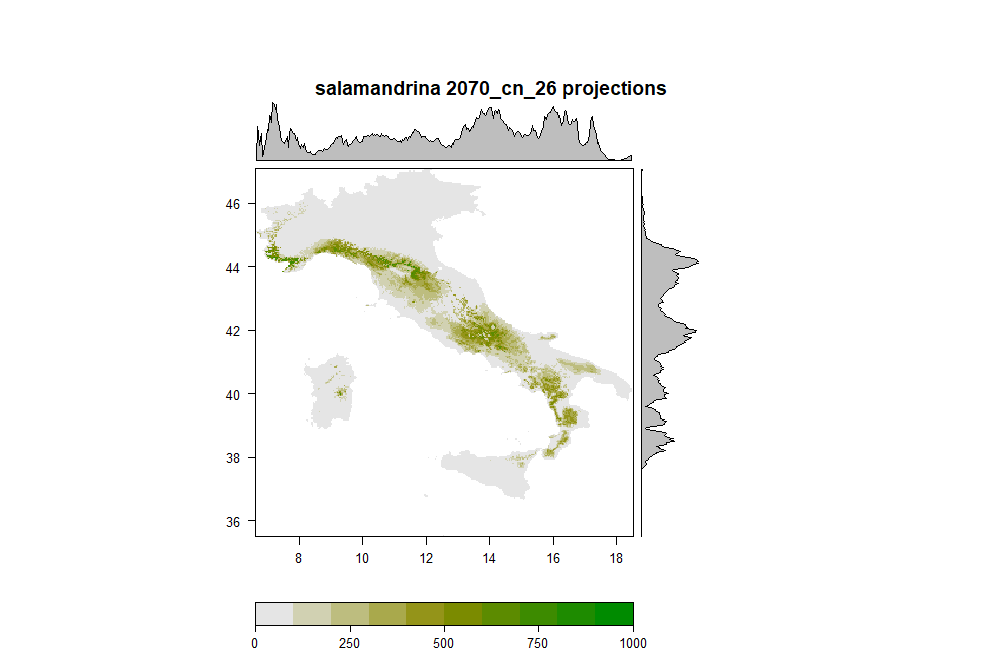


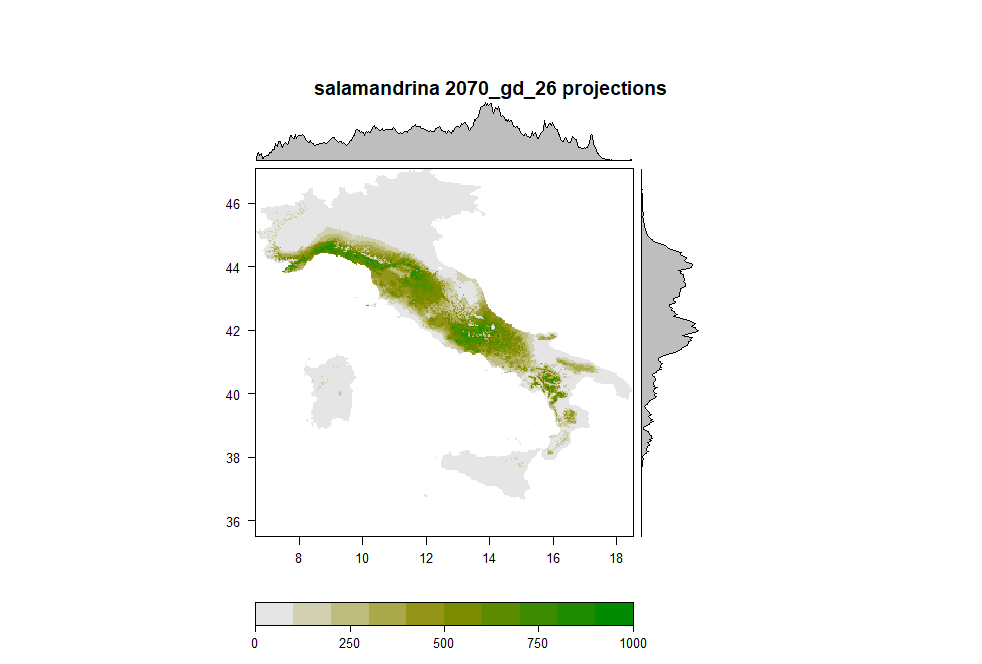

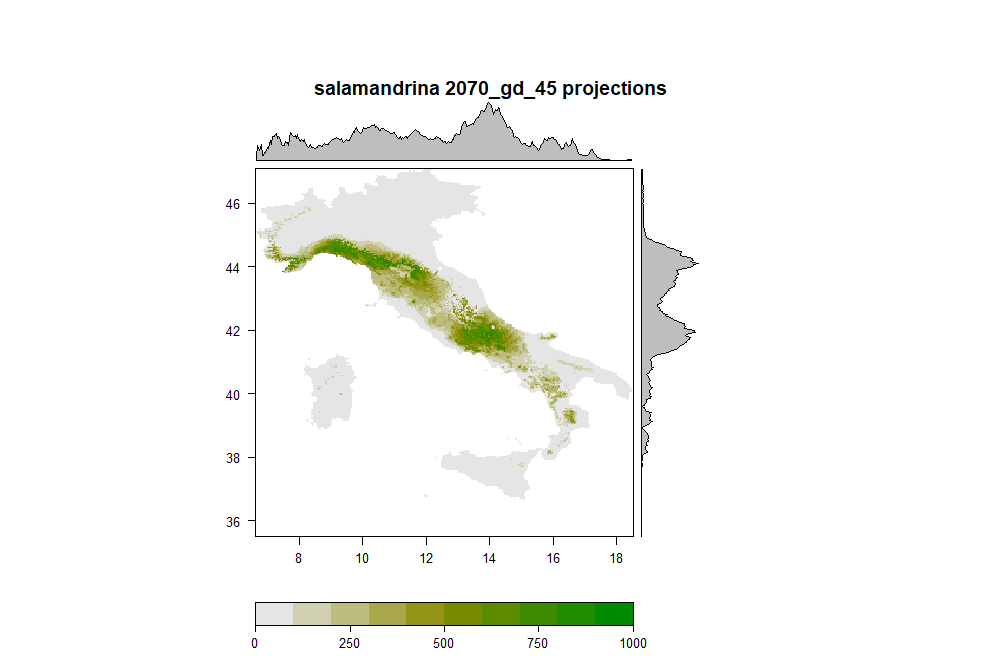

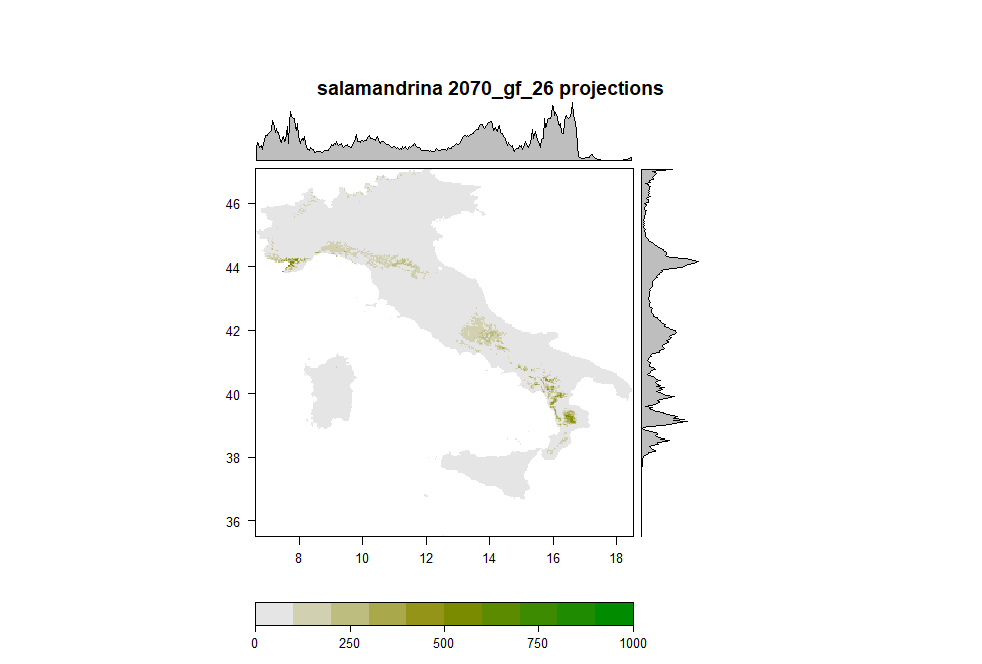


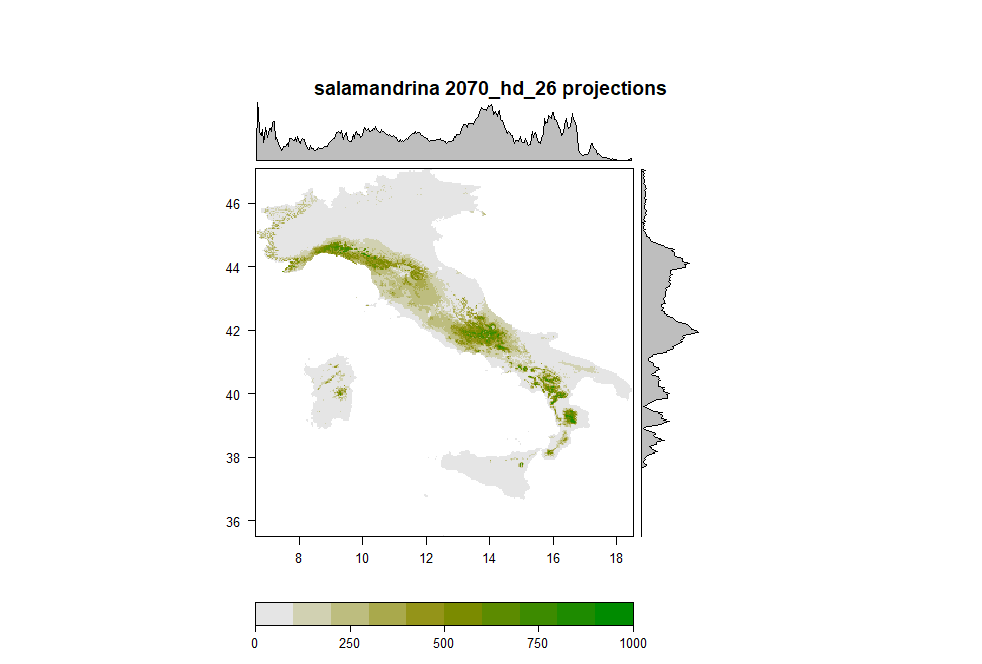

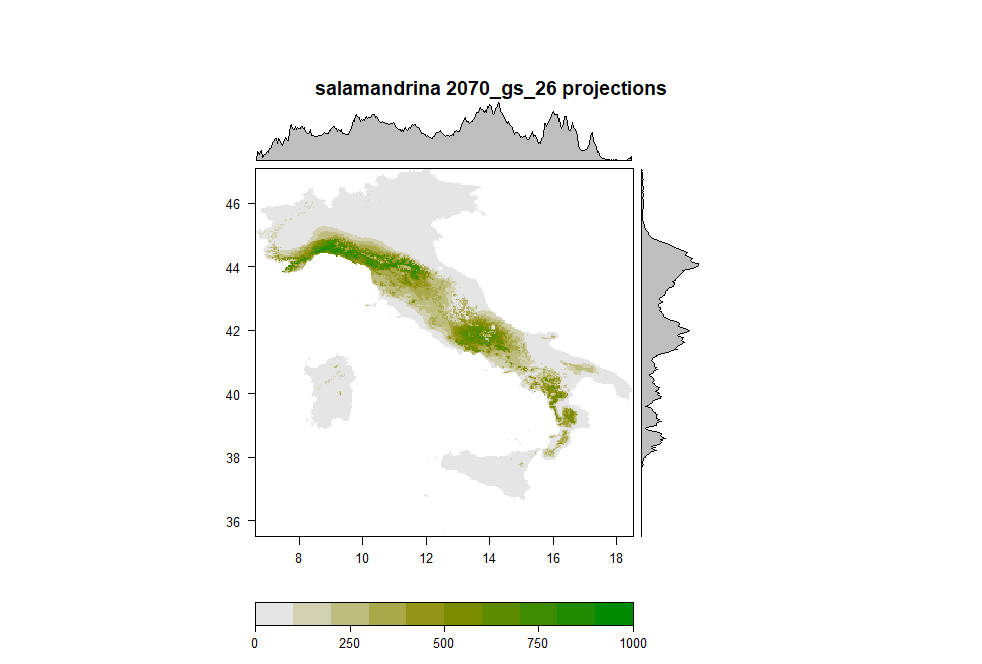

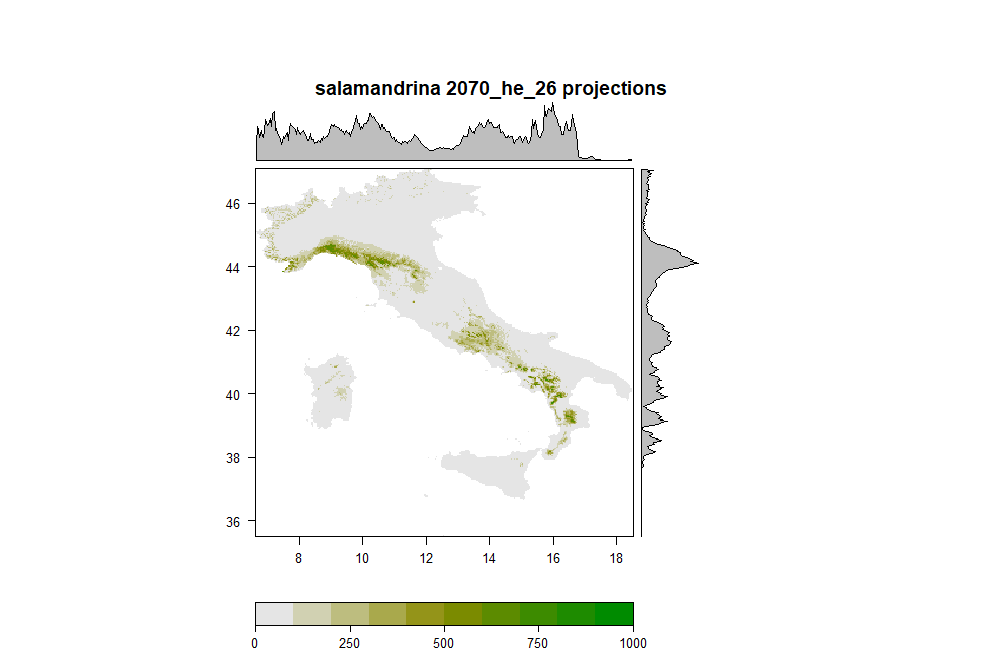

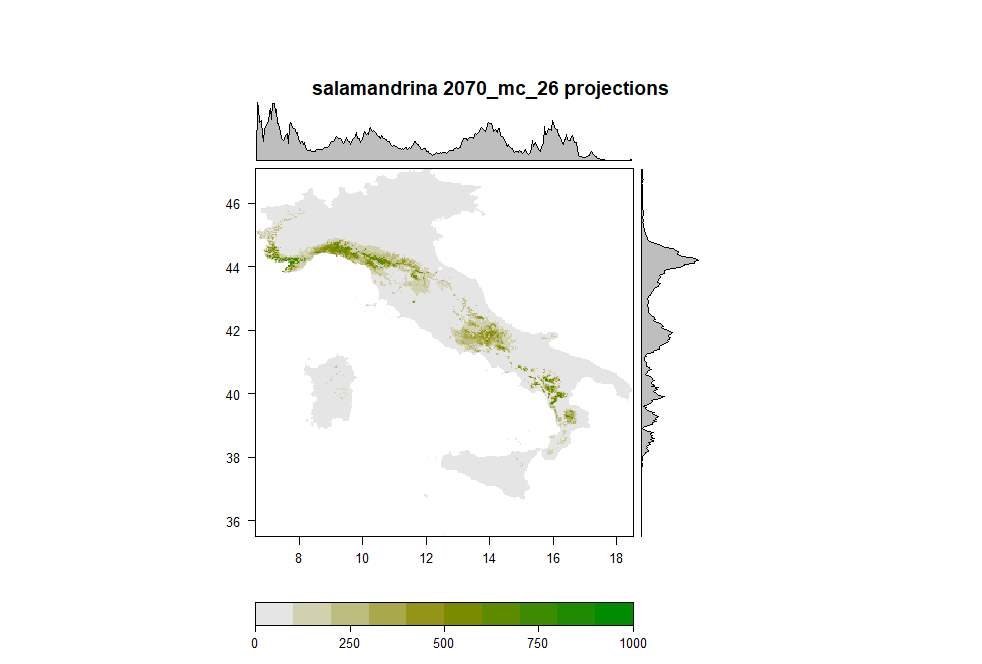

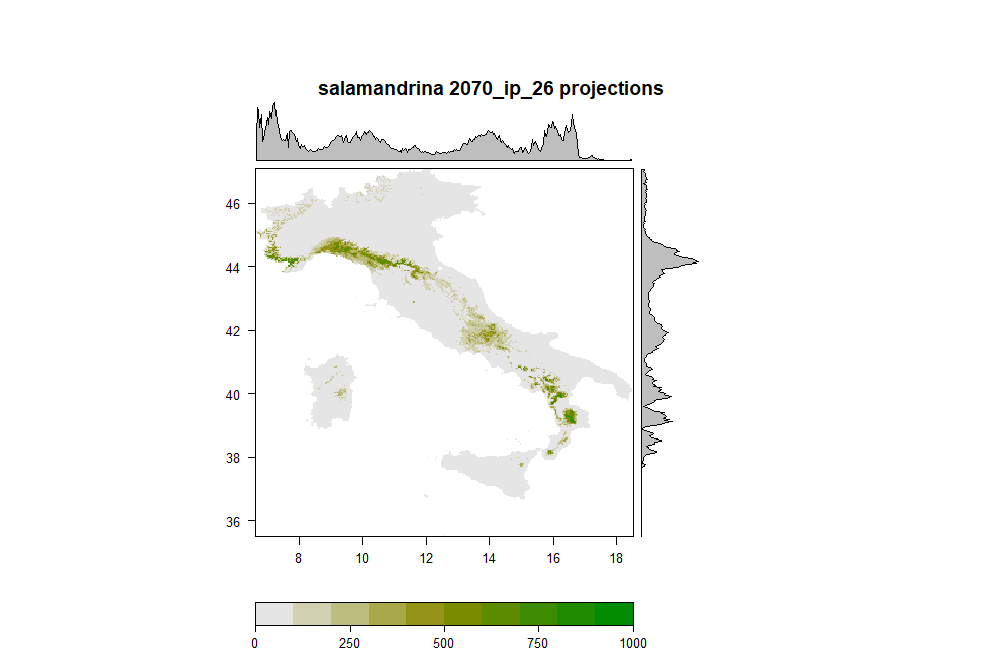

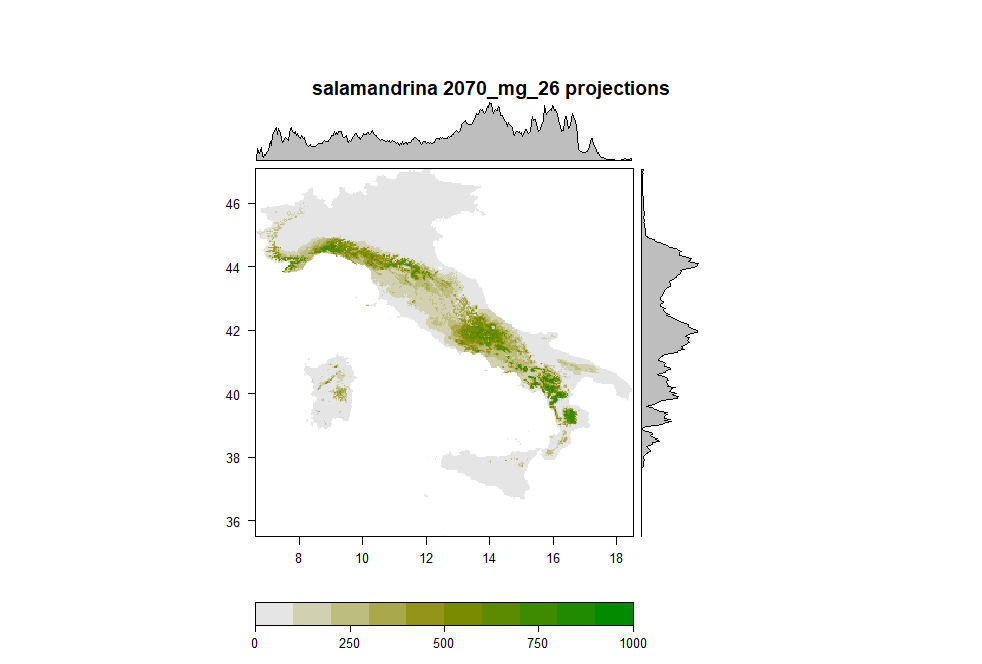


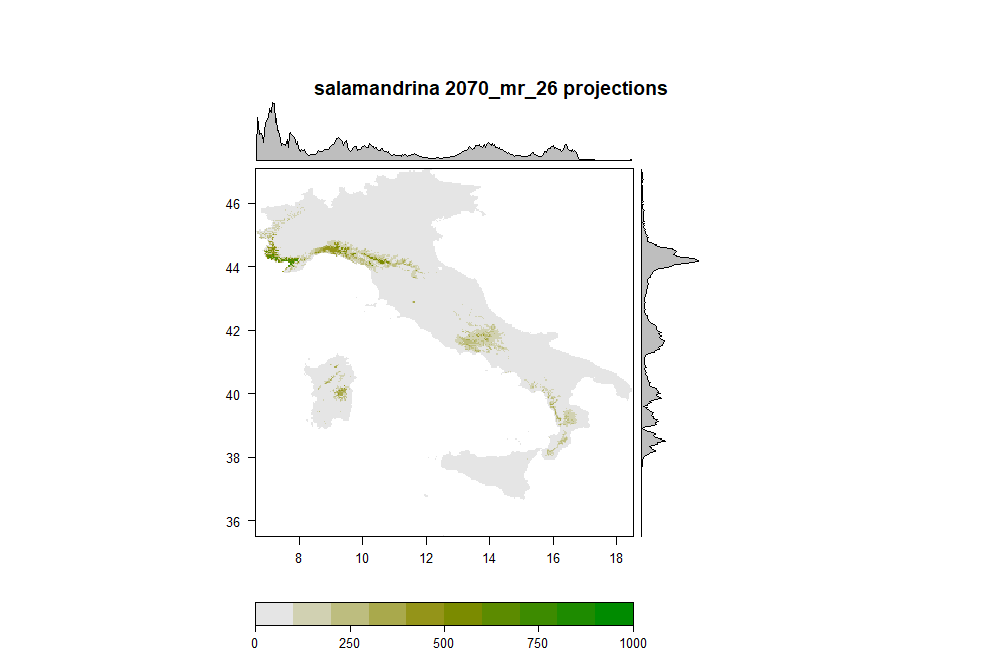

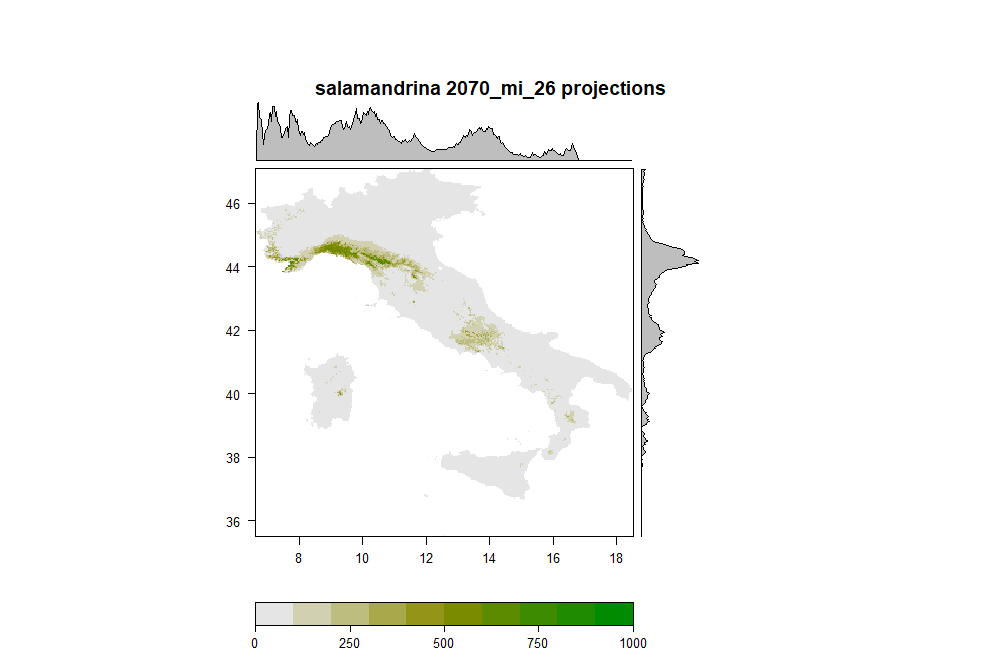

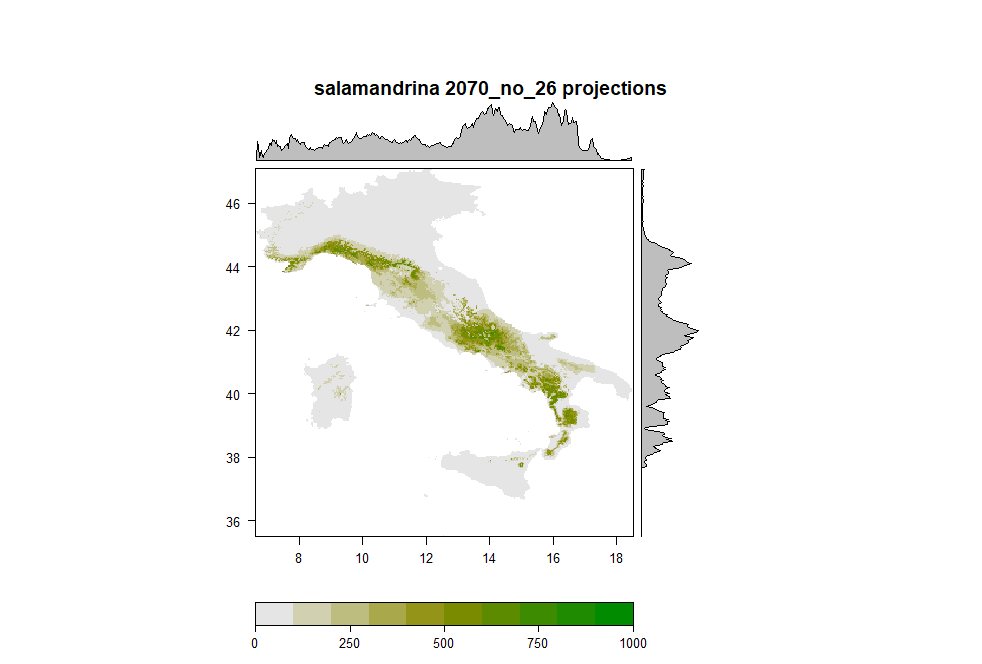


**Appendix S5. Ensemble model evaluation obtained through R package ‘biomod2’**.

|  | salamandrina_EMmeanByTSS_mergedAlgo_mergedRun_mergedData.Testing.data | salamandrina_EMmeanByTSS_mergedAlgo_mergedRun_mergedData.Cutoff | salamandrina_EMmeanByTSS_mergedAlgo_mergedRun_mergedData.Sensitivity | salamandrina_EMmeanByTSS_mergedAlgo_mergedRun_mergedData.Specificity |
| --- | --- | --- | --- | --- |
| TSS | 0.977 | 533 | 98.843 | 98.875 |

**Appendix S6.** Histograms showing the distribution of *Salamandrina* versus different bioclimatic variables (from WorldClim2.1). Temperatures are expressed in Celsius degrees, and precipitation is measured in mm. Temperature and precipitation seasonality are as defined in Appendix S1.


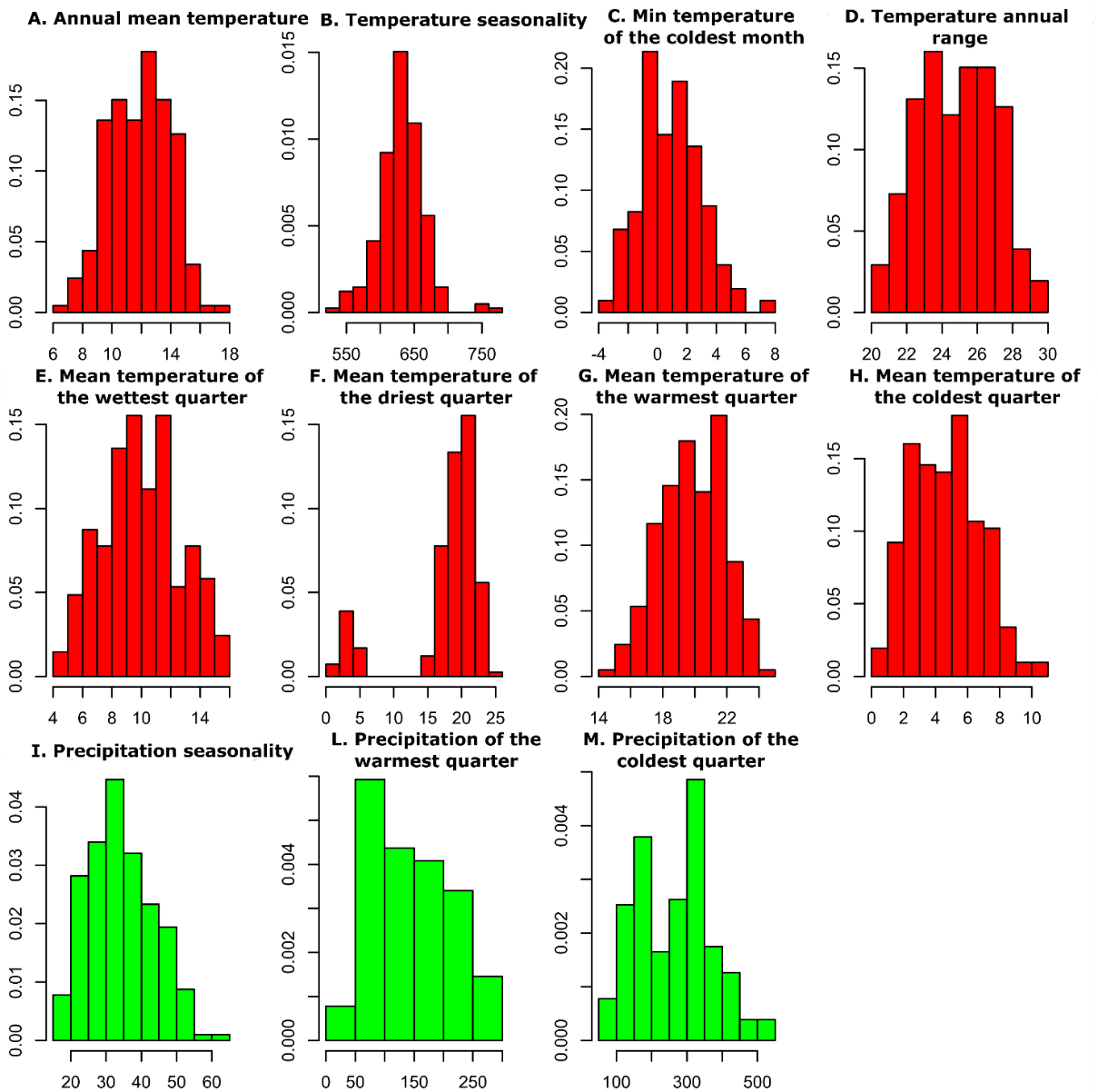


**Appendix S7.** List of contributors of presence data of *Salamandrina* spp. to the "Commissione Atlante" of the *Societas Herpetologica Italica*:

Gaspare Adinolfi

Alessandro Ammann

Guido Baldi

Rosario Balestrieri

Daniele Baroni

Remo Bartolomei

Angelo Battaglia

Franco Bernini

Domenico Bevacqua

Emanuele Biggi

Alex Borrini

Andrea Boscherini

Adam Bouderka

Stefano Brigidi

Giacomo Bruni

Fabrizio Bulgarini

Giovanni Capobianco

Elena Cecchinelli

Stefano Celletti

Carlo Ciani

Luca Colonnelli

Gianluca Congi

Luca Coppari

Michele Coppola

Andrea Costa

Renato Cottalasso

Federico Crovetto

Anna Rita Di Cerbo

Matteo di Nicola

Stefano Doglio

Dario Domeneghetti

David Fiacchini

Giovanni Fontanesi

Carlo Fracasso

Egidio Fulco

Ilaria Guj

Alfonso Iorio

Arnaldo Iudici

Sara Lefosse

Giorgio Leoni

Cristiano Liuzzi

Guglielmo Londi

Enrico Lunghi

Paolo Malenotti

Antonio Mancuso

Raoul Manenti

Giovanni Marcantonio

Maurizio Marrese

Fabio Mastropasqua

Francesca Montioni

Marco Morbidelli

Sergio Muratore

Moreno Nalin

Veronica Nanni

Nicola Norante

Tommaso Notomista

Dario Ottonello

Mario Pellegrini

Emanuela Peria

Fabrizio Petrassi

Jerry Pieri

Lucia Pizzocaro

Ranieri Raimondi

Edoardo Razzetti

Stefano Risa

Giuseppe Romeo

Diego Rubolini

Andrea Russo

Dario Salemi

Roberto Santopaolo

Bruno Santucci

Stefano Sarrocco

Valerio Sbordoni

Silvia Sgrosso

Roberto Sindaco

Adrien Sprumont

Giovanni Talpone

Stefano Tito

Domenico Verducci

Marco Zuffi
